# Direct Acting Antivirals for the Treatment of Chronic Viral Hepatitis

**DOI:** 10.6064/2012/478631

**Published:** 2012-11-25

**Authors:** Peter Karayiannis

**Affiliations:** Section of Hepatology and Gastroenterology, Department of Medicine, Imperial College, St Mary's Campus, London W2 1PG, UK

## Abstract

The development and evaluation of antiviral agents through carefully designed clinical trials over the last 25 years have heralded a new dawn in the treatment of patients chronically infected with the hepatitis B and C viruses, but not so for the D virus (HBV, HCV, and HDV). The introduction of direct acting antivirals (DDAs) for the treatment of HBV carriers has permitted the long-term use of these compounds for the continuous suppression of viral replication, whilst in the case of HCV in combination with the standard of care [SOC, pegylated interferon (PegIFN), and ribavirin] sustained virological responses (SVRs) have been achieved with increasing frequency. Progress in the case of HDV has been slow and lacking in significant breakthroughs.This paper aims to summarise the current state of play in treatment approaches for chonic viral hepatitis patients and future perspectives.

## 1. Introduction

Conservative estimates of the number of individuals worldwide who are thought to be chronically infected with either HBV or HCV are placed at over 350 [1] and 200 [2] million, respectively. It has long been established through epidemiological surveys that these patients are at increased risk of developing cirrhosis, hepatic decompensation, and hepatocellular carcinoma (HCC). About 1 million people die per year as a result of HBV-related liver pathologies [3]. In resource-limited countries, HBV infection accounts for 30% of cirrhotic patients and 53% of those with HCC [4]. On the other hand, HCV is responsible for approximately 350000 deaths every year [5]. The only means of preventing these un-necessary deaths is therapeutic intervention through the use of immune modulators and direct acting antivirals (DDAs). The ultimate goals of treatment are to achieve a sustainable suppression of replication and remission of liver disease in the case of HBV, and complete eradication of the virus from the liver in the case of HCV. 

For many years, the only choice for treatment was interferon alpha (IFN*α*), lymphoblastoid initially and recombinant subsequently, both of which have more recently been superceded by the pegylated form (PegIFN), which requires intramuscular injection only once a week as opposed to three times a week with the previous forms. Interferon has not only immunomodulatory, but also antiproliferative and antiviral effects. It acts by promoting cytotoxic T-cell activity for lysis of infected hepatocytes and by stimulating cytokine production for control of viral replication.

DDAs on the other hand constitute a more recent development based on increasing knowledge of the molecular biology of the hepatitis viruses. In the case of HCV, the resolution of the 3-dimensional structure of important viral enzymes such as the NS3 serine protease and the RNA-dependent RNA polymerase (RdRp), and the in vitro models of viral replication that have allowed the study of virus entry, replication, morphogenesis, and identified host factors that are required for this process, have been invaluable in the design and testing of drugs under development. Such drugs act directly as viral lifecycle inhibitors. The current choices of treatment will be reviewed in turn for each virus, as well as results from current clinical or preclinical trials with other agents in development, and which most likely will truly revolutionise future treatment approaches.

## 2. Hepatitis B Virus

### 2.1. Virology

 HBV is the prototype virus of the *Hepadnaviridae*, a name which signifies the hepatotropism and DNA nature of the genome of its members. The mature virion or Dane particle measuring 45 nm in diameter is spherical in nature and consists of an outer envelope comprised of the hepatitis B surface proteins (HBsAg) in a lipid bilayer derived from the host. The envelope encloses the nucleocapsid of the virus which is composed of the self-assembling core protein (HBcAg). This in turn encloses the viral genome which is a relaxed circular, partially double stranded DNA molecule of 3.2 kb in length. All of the nucleotide sequence of the genome is organised in 4 partially or totally overlapping open reading frames (ORFs), [6] which are transcribed with the help of two enhancer elements and four promoters within the genome. The Pre-S/S ORF encodes the three envelope glycoproteins which are known as the large (L), middle (M), and small (S) HBsAgs. All three proteins share the S domain, whilst the L (PreS1+PreS2+S) and M (PreS2+S) proteins have N-terminal extensions as indicated [7]. The S domain contains the major hydrophilic region known as the *α* determinant which confers group specificity, a cluster of B-cell epitopes between amino acid positions 90–160. This constitutes the main target of neutralising antibodies, both natural and vaccine induced [8]. In addition, subviral particles in the form of 22 nm spheres and filaments composed entirely of HBsAg are released into the circulation in numbers up to a million in excess of the infectious Dane virions [7].

The amino acid sequence of the S protein has allowed the identification of at least 8 genotypes of the virus, the most common of which are genotypes A–F. 

Genotype A is seen mostly in Northern Europe, whilst D in Southern Europe, the Middle East, and Indian subcontinent [9, 10]. Genotypes B and C are prevalent in the Far East, whilst genotypes E and F are found in Africa and South America, respectively [11]. The predominant genotypes in The United States are A and C [12].

The precore/core ORF encodes for two translation products, namely, the longer precore polypeptide initiated at the first AUG and which constitutes the precursor of the soluble hepatitis B e antigen (HBeAg), and the core protein or HBcAg. The synthesis of the latter utilises the second in frame AUG of the ORF. The HBeAg is formed by proteolytic cleavage of its N-terminal 19 amino acids which constitute a signal peptide and truncation of its C-terminus, through the action of peptidases within the lumen of the endoplasmic reticulum (ER) network resulting in its secretion. HBeAg is thus a nonstructural protein, not essential for viral replication, a marker of infectivity, and with tolerogenic and immune modulating activity that plays a significant role in viral persistence. The shortest ORF encodes for the X protein which is essential for viral replication and has transactivating potential. The longest ORF is that for the polymerase, which has four domains; the N-terminus is occupied by the terminal protein which is involved in priming DNA synthesis followed by the spacer region, then the reverse transcriptase (rt)/DNA polymerase domain, and finally the RNAse H domain at the C-terminus. All RNA transcripts involved in the translation of these proteins are coterminal, polyadenylated, and capped [6, 7, 13].

### 2.2. Replication

The hepatocyte receptor responsible for virus attachment remains unknown to this date. In contrast, amino acid positions 21–47 of the Pre-S1 have been implicated in virus binding to the hepatocyte membrane [14, 15]. A domain within S may assist in this process by bringing the virion in close contact with the cell membrane, and thus facilitating the specific interaction of the Pre-S1 domain with its receptor [16]. Following internalisation the virion is uncoated in the cytosol, the naked core particles are trafficked to the nuclear pore through which the genome penetrates into the nucleoplasm, where it is converted into a double-stranded covalently closed circular DNA (cccDNA) molecule, following removal of the covalently bonded terminal protein from the negative (−)-DNA strand and repair of the nick, as well as completion and ligation of the shorter positive (+)-strand [6, 7, 13, 17]. Intrahepatic cccDNA load ranges from 0.1–1 copy per cell, or 10–1000 copies per infected cell, depending on the HBeAg status of the patient as explained below. cccDNA remains in episomal form and in its transcriptionally active state associates with histones and other proteins, and through recruitment of a number of liver specific transcription factors serves as the template for viral transcript synthesis by host RNA polymerase II. Most antiviral agents so far have been unable to prevent the replenishment of the cccDNA pool from genonic HBV-DNA recycled from immature core particles in the cytoplasm to the nucleus, or to radically eliminate cccDNA-containing hepatocytes [18]. This persistence explains the rather rapid rebound in serum HBV-DNA after cessation of antiviral treatment and the reactivation of HBV infection following immunosuppression of individuals in spite of the presence of immune clearance markers.

**Figure 1:**
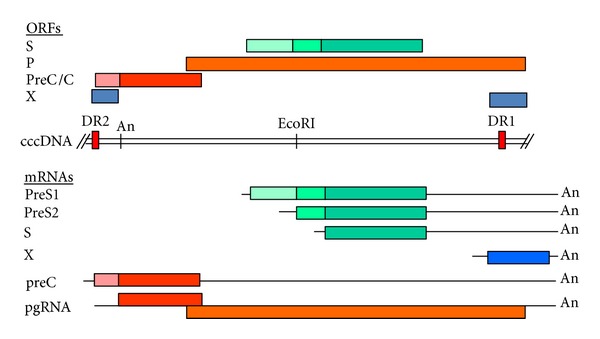
The closed covalent circular DNA of HBV depicted in linear form and showing positions of the direct repeats (DR) 1 and 2, and the polyadenylation signal (An). The open readings frames encoding for the surface (S), polymerase (P), X and Precore/core proteins are shown above, whilst the RNA transcripts are shown below the cccDNA. The various RNA transcripts terminate at the common polyadenylation signal.

**Figure 2:**
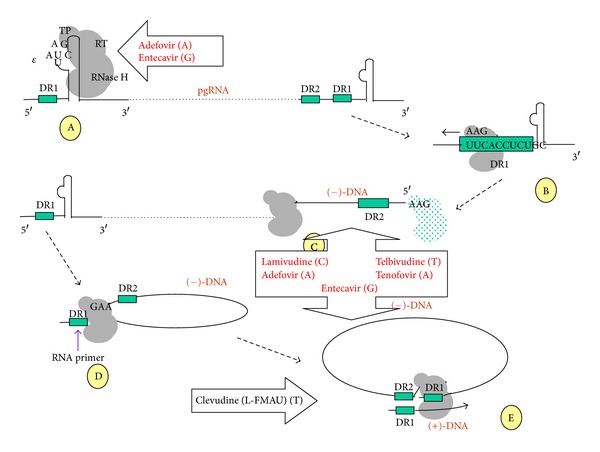
The replication strategy of hepatitis B virus and stages of: (A) binding of the viral polymerase to *ε* and synthesis of a short primer using as template the nucleotide sequence of the bulge as shown. (B) Translocation of the polymerase/primer complex to the 3′ end of the pgRNA and base-pairing with DR1. (C) (−)-DNA strand synthesis and degradation of the pgRNA template by the RNase H domain of the polymerase, apart from its terminal 18 or so bases. These bases constitute the RNA primer which initiates (+)-DNA strand synthesis (D). Translocation of the primer to the 5′ end of the newly synthesised (−)-DNA strand and annealing with the homologous DR2 region leads to (+)-DNA strand synthesis. This proceeds in the direction shown by the arrow, which necessitates yet another translocation event to the 3′ end of the (−)-DNA strand. Both of these events are most likely facilitated by the effective circularisation of the (−)-DNA strand. This becomes possible, as a result of the covalent attachment of the 5′ end of the strand to the polymerase, which is maintained during continued synthesis of the (−)-DNA strand. Thus, the two ends of the strand are brought into close proximity with each other (E). Points of action of nucleos(t)ide analogues are shown by open arrows.

The pregenomic RNA (pgRNA) is one of two RNA transcripts longer than genome length (3.5 kb), which is the template for (−)-DNA strand synthesis as well as the message encoding both the core and polymerase proteins (Figures 1 and 2) [19]. The polymerase engages epsilon (*ε*), a secondary RNA structure at the 5′ end of the pgRNA, [20] triggering encapsidation of the complex by the core protein. Thus subsequent steps in virus nucleic acid replication take place within the nucleocapsid [6, 7, 13, 21]. As a consequence of the terminal redundancy of the pgRNA, the epsilon sequence and flanking region containing direct repeat 1 (DR1) are duplicated (Figure 2). The bulge of the *ε* structure serves as a template for the synthesis of a 3 nucleotide long DNA primer, which is covalently attached to the polymerase though a tyrosine residue of the terminal protein (position 96) [22, 23]. This event involves the *ε* structure at the 5′ end of the pgRNA, and is then followed by the translocation of the polymerase-primer complex to the 3′, where the primer hybridises to a homologous region of DR1. This in turn initiates (−)-DNA strand synthesis by reverse transcription as the complex proceeds towards the 5′ end of the pgRNA. Concurrently, the RNA template is degraded by the RNAse H activity of the polymerase, except for the final 18 or so ribonucleotides. A second translocation event then occurs during which the ribonucleotide primer hybridises with the DR1 region at the 5′ end of the newly synthesised (−)-DNA strand. A template exchange occurs that allows the (+)-DNA strand synthesis to proceed along the 5′ end of the complete (−)-DNA strand, effectively circularising the genome [21]. Pores on the surface of the capsid facilitate nucleotide entry for DNA strand synthesis, including nucleos(t)ide analogues during treatment [24]. Envelopment of the mature nucleocapsid by budding through the ER membrane, [25] leads to exhaustion of the nucleotide pool within the capsid leaving the (+)-DNA strand incomplete.

### 2.3. Mutations

Natural stable variants of the virus give rise to well-recognised serological subtypes and genotypes [26]. However, HBV has a higher mutation rate than other DNA viruses (2 × 10^−4^) base substitutions per site per year), [27] through error prone steps in the replication cycle of the virus. These may occur during pgRNA synthesis by the cellular RNA polymerase II, as RNA polymerases show inherently low copying fidelity, but also during reverse transcription due to the lack of proof reading capacity by the viral polymerase. Fluctuations in the composition of the intracellular nucleotide pools are another possible contributing factor. A lot of these mutations are lethal to the virus, but those which offer it a replication advantage, facilitate immune escape, or cause resistance to antiviral drugs, as explained later, can be preferentially selected. 

A G1896A substitution in the precore region creates a premature termination codon (precore stop-codon variant) that abrogates HBeAg production [28–30]. The variant is seen in genotype D which prevails in the Mediterranean basin, Middle East, and Indian subcontinent, genotypes B and C in countries of the Far East, and genotype E in Africa. This mutation is rarely detected in genotype A strains found in Northern Europe and North America. A double mutation in the core promoter region (A1762T, G1764A) [31] downregulates transcription of the precore mRNA encoding the HBeAg precursor protein, whilst pgRNA synthesis is upregulated [32]. These variants predominate in HBeAg negative patients with detectable levels of HBV-DNA, [33] may cause frequent exacerbations with fluctuating transaminase levels, [34–36] and have important implications in the treatment of such patients with antiviral agents [34]. 

### 2.4. Patient Groups

The main routes of HBV transmission are not only perinatal, percutaneous, and sexual, but also by contact with open cuts and sores, as may occur between children in hyperendemic areas [37]. The risk of progressing to chronicity following acute infection is age dependent. Over 90% of newborns to HBeAg positive mothers, 25–30% in infants and very young children, and around 5% of adults are at risk of becoming chronic carriers after exposure, [38–40] defined as the persistence of HBsAg in serum for longer then six months [41]. The natural history of chronic hepatitis B (CHB) may progress through four phases which in turn are the immune tolerant, the immune clearance, the inactive or nonreplicative, and the reactivation or immune escape phases. These phases do not occur in all individuals and this may depend on the age and route of exposure and may not be sequential [42]. 

The immune tolerant phase is normally found in patients who are exposed to the virus at a young age and is characterised by HBeAg positivity, high HBV-DNA levels (>20000 IU/mL), normal or near normal ALT levels, normal/minimal histological activity, and HBsAg levels ranging from 4.5-5 log IU/mL. This phase may last for 20–30 years [43–46]. In contrast, this phase in those exposed to the virus in adulthood is short and leads to the immune clearance phase which is characterised once again by HBeAg positivity, elevated serum ALT levels associated with histological damage, fluctuating HBV-DNA levels, and lower levels of HBsAg (4.3 log IU/mL) [47, 48]. During this period the virus comes under immune attack with emergence of the precore and core promoter mutants. This sequence of events culminates in HBeAg seroconversion to anti-HBe that leads into the immune control or nonreplicative phase with extremely low or undetectable HBV-DNA and normal ALT levels. A subset of patients however, for reasons that remain unknown reactivate viral replication forming a group that is referred to as the HBeAg-negative CHB, characterised by HBV-DNA levels >2000 IU/mL, fluctuating ALT levels once again, liver damage and higher levels of HBsAg (3.5 log IU/mL) than during the immune control phase [48]. The patients in the phases of immune clearance and immune escape are the most likely candidates for antiviral treatment with currently approved drugs, including difficult to treat groups such as cirrhotics, immunosuppressed patients as a consequence of organ transplantation and patients with HIV infection.

## 3. Desirable End Points of Treatment

Successful antiviral treatment relies on the achievement of the following biochemical, virological, and histological end-points. These are normalisation of ALT levels, suppression of HBV-DNA to undetectable levels monitored by real-time PCR assays with a lower limit of detection of 10–20 IU/mL (both in HBeAg-positive and-negative CHB), loss of HBeAg with or without development of anti-HBe, and a decrease in the necroinflammatory score by ≥2 points with no worsening in fibrosis. Loss of HBsAg with or without development of anti-HBs is a more desirable outcome but not so easily achievable. Spontaneous HBsAg loss and/or seroconversion is a rare event in CHB occurring at an annual rate of around 1% [49].

## 4. Pegylated Interferon (PegIFN)

Although IFN is not a DAA, for the sake of completion and comparative purposes will be dealt with here in brief. Recombinant or standard IFN licensed for the treatment of CHB nearly two decades ago has been replaced with PegIFN *α* 2a (Pegasys) with improved pharmacokinetics, more potent and once-a-week subcutaneous injection only as opposed to 3 of its prototype. Two phase III studies compared PegIFN to lamivudine (LMV, a nucleoside analogue) or a combination of both for 48 weeks in HBeAg-positive and-negative patients [50, 51]. In the former, PegIFN with or without LMV was shown to be superior to LMV monotherapy, [50, 52, 53] although a higher log reduction of HBV-DNA was observed with the combination at 48 weeks but not at the end of followup (2-4.5 versus 4-7.2 copies/mL) [50, 52]. Long-term followup of European and Far Eastern patients indicated that 19% had undetectable levels of HBV-DNA, HBeAg loss had occurred in 37%, and more importantly 11% had lost HBsAg after a mean of 3.5 years after cessation of treatment. There were differences though depending on genotype; HBsAg loss was significantly higher in genotype A than non-A infection (28% versus 3%). What is more, among initial responders durable loss of HBeAg was recorded in 80% of patients and HBsAg loss in 30% of them. This is in contrast to the 2.4% of HBsAg loss seen after 5 years in patients with genotypes B and C from Hong Kong, with similar levels of durable HBeAg loss [54]. Other than genotype, a high level of ALT and a low level of HBV-DNA appear to be good predictors of response [50, 52].

In the case of HBeAg negative patients, similar studies showed after 48 weeks of treatment in patients who received PegIFN alone or in combination with LMV had a significantly higher rate of sustained, off-treatment responses, which were maintained when the patients were reevaluated 3 years after stopping treatment [51, 55, 56]. Younger age, female sex, a high level of ALT, a low level of HBV-DNA, and genotypes B and C have been shown to be associated with a favourable response to treatment [57].

More recently, quantitative measurement of HBsAg levels during treatment has been shown to be a useful tool in monitoring PegIFN treatment efficacy. An on treatment decline of HBsAg of >1 log IU/mL at 24 weeks of treatment had a high predictive value of a sustained response [48, 58]. Moreover, having an HBsAg level of <10 IU/mL at the end of therapy and a 1 log IU/mL drop in levels during treatment were strong predictors of durability of response and loss of HBsAg 3 years after treatment discontinuation [59]. 

## 5. Nucleos(t)ide Analogues (NAs)

Currently five NAs are licensed for the treatment of CHB. These include in chronological order of licensing LMV, adefovir dipivoxil (ADV), entecavir (ETV), telbivudine (TBV), and tenofovir disoproxil fumarate (TFV). NAs act by suppressing HBV replication at the level of DNA synthesis, and this because they are chemically synthesised drugs which are able to mimic natural nucleos(t)ides. As such, they are incorporated into newly synthesised HBV-DNA causing chain termination (Figure 2), and thus inhibiting viral replication. In addition, some of them competitively inhibit the DNA-dependent and reverse transcriptase activity of the viral polymerase. For this to occur, the NAs need to be phosphorylated within cells to their triphosphate counterparts and possible steps in the life cycle of HBV that may be inhibited include the synthesis of the (−)-DNA strand by reverse transcription and that of the (+)-DNA strand, as well as the synthesis of the primer for initiation of DNA synthesis (Figure 2). Experiments in woodchucks suggest that nucleoside analogue treatment does not have an appreciable effect on the cccDNA pool in hepatocytes [60]. 

### 5.1. Lamivudine (LMV)

Lamivudine is the L-enantiomer of the deoxycytidine analogue 2′,3′-dideoxy-3′-thiacytidine (3TC) and was the first NA to be approved for the treatment of CHB (1998) [61, 62]. Widely used when first licensed, LMV is not as widely used nowadays in view of the newer NAs with a better genetic barrier record to resistance. Since LMV acts by terminating viral DNA synthesis [62, 63] and competitively inhibiting the viral polymerase/rt, [63] it is equally effective in patients of any race, but also against both the wild-type virus and precore/core promoter variants [56, 64–67]. LMV is administered orally and the recommended dose for adults is 100 mg per day. LMV is still in use in poor resource countries.

Several randomised clinical trials of LMV monotherapy in HBeAg-positive patients showed that 1 year of treatment induced HBeAg seroconversion in 16–18% of them, compared to 4–6% in controls [68–70]. Histologic improvement by at least 2 points in the histological activity score was observed in 49–56% and 25% of treated and control patients, respectively. HBeAg seroconversion rates increased with length of therapy rising from 17% at 1 year to 27%, 36%, and 47% at years 2, 3 and 4 respectively [71–73]. The durability of HBeAg seroconversion in lamivudine treated patients is variable ranging in some studies between 38–73%, [72–74] but can be consolidated by extending treatment for 6 months or more [75, 76].

LMV treatment of HBeAg negative patients achieved HBV-DNA undetectability in up to 72% of patients and ALT normalisation in 65–96% of patients at the end of treatment [56, 64, 77–83]. Similarly, there was a beneficial impact on the histological picture in 60% of patients, and slow down or even improvement in the fibrosis score in 11–35% of patients [56, 83, 84]. The arrest or reduction in the immune-mediated inflammatory response in the liver and the reduction in scarring, not only benefited the fibrosis score, but also resulted in a decrease in the incidence of HCC [85–87]. Patient relapse rates after 1 year of followup amounted to 45–74% (sustained response of only 11–20%), hence the recommendation for long-term use in these patients and in HBeAg positive patients not achieving HBeAg loss. The efficacy of LMV for treatment periods longer than 12 months in HBeAg negative patients indicated drops of 14–36% by 24 months in comparison to those at 12 months [64, 79, 80, 88]. 

The most important predictor of a favourable response following LMV treatment is the pretreatment ALT level [89, 90]. The major drawback of LMV is the high rate of emergence of drug resistant variants that can lead to virological and biochemical relapse. Breakthrough infections have been recorded in 27% of HBeAg-positive patients after 1 year of treatment, [68–70] increasing to 38%, 49%, and 66% for years 2, 3, and 4, and up to 76% at year 8, respectively [71–73, 87]. Lamivudine resistant variants also arise in HBeAg-negative patients ranging from 27% at 1 year to 70–80 by year 5 [56, 64, 78, 80]. Emergence of the lamivudine resistant variants may be accompanied by acute exacerbation of liver disease [91, 92]. Moreover, HBeAg seroconversion has been reported to occur in about a quarter of the patients with breakthroughs, who continue treatment [71, 91]. 

The rt/DNA polymerase domain contains at least 5 subdomains (A–E) which are spatially separated, but closely associated with the normal function of the protein. Subdomain C contains the characteristic YMDD (tyrosine-methionine-aspartate-aspartate) motif of the catalytic site [93]. LMV resistance is associated with amino acid substitutions primarily in subdomain C and may be accompanied by others in subdomain B [92, 94, 95]. Substitutions in subdomain C affect the YMDD motif, and include rtM204I (YIDD) and rt M204V (YVDD) [69, 73, 94, 96–100]. Viruses with these mutations are less replication fit than the wild-type virus [101, 102]. It is postulated that an rtL180 M mutation in subdomain B accompanying the rtM204V change, [94, 97, 99, 100] restores replication competency, [102] as rtV173L also in subdomain B may do so also [92, 103, 104]. One other mutant, rtA181T/V has been shown to be resistant to LMV following prolonged treatment, [105] with cross-resistance to ADV [106].

### 5.2. Adefovir Dipivoxil (ADV)

Adefovir (Hepsera) or bis-pivaloyloxymethyl-9-(2-phosphonyl-methoxyethyl) adenine (PMEA) is an NA of adenosine monophosphate and was approved in 2002 for the treatment of CHB (10 mg/day). Adefovir inhibits HBV replication, [107, 108] but appears to be less potent than other NAs with regard to HBV-DNA reduction. Its efficacy has been assessed in the clinical setting, and has been shown to be active against LMV resistant mutants [109–116]. Treatment for one year led to an HBeAg seroconversion rate of 12%, whilst histological improvement was seen in 53% of HBeAg positive patients [117]. HBeAg serconversion was sustained in 91% of these patients [76, 118]. ADV is also effective in HBeAg negative patients with 51% and 64–69% achieving HBV-DNA undetectability and histological improvement, respectively [119]. Prolonged treatment leads to emergence of resistance with two main mutations; rtA181T/V and rtN236T [120]. Genotypic resistance is less frequent than LMV with an incidence of 0%, 3%, 11%, 18% and 29% after 1, 2, 3, 4, and 5 years of treatment, respectively, in HBeAg negative patients [121]. ADV has now been superceded by TFV use instead.

### 5.3. Entecavir (ETV)

Entecavir is a nucleoside analogue of adenosine monophosphate which was licensed for the treatment of CHB in 2005. It is superior to LMV and is used at a dose of 0.5 mg/day and 1 mg/day for treatment naïve and LMV resistant patients, respectively. It has a potent antihepadnaviral activity, [122–124] as demonstrated against both HBeAg positive and negative patients. ETV was compared to LMV in treatment naïve HBeAg positive patients after 48 weeks of treatment and showed a 6.98 versus 5.4 log drop in HBV-DNA in copies/mL [125]. In another trial, treatment for 52 weeks resulted in a reduction in HBV-DNA to <400 IU/mL, histological improvement, and normalisation of ALT levels in 67% versus 36%, 72% versus 62%, and 78% versus 70%, compared to LMV treated patients once again [126]. HBeAg seroconversion rates however were very similar at 21% versus 18%. Similar results were obtained in a trial comparing ETV to ADV [127].

Similar results were obtained in HBeAg negative patients with indefinite treatment when comparing ETV to LMV; HBV-DNA suppression was recorded in 91% versus 73% and histological improvement in 70% versus 61% [128, 129]. After 5 years of treatment ETV maintained HBV-DNA suppression at levels <300 copies/mL in 94% of patients, [130] whilst 84% of patients had improvements in the their fibrotic scores after 6 years of treatment [131].

HBsAg loss was seen in 1.7% of ETV versus 1.1% of LMV patients treated for 48 weeks, rising to 5% versus 3% at week 96 [132]. The probability of this happening appears to be higher in genotype A and D infected male patients of Caucasian race, with early HBeAg seroconversion. In the case of HBeAg negative patients, ETV does not appear to have an impact on HBsAg loss [133].

ETV has a high genetic barrier with an extremely low incidence of resistance [134]. In treatment naïve patients, no resistance was detected by 2 years of treatment [135] and only 1.2% at 5 years [130, 134]. Resistance was shown to arise in patients with preexisting LMV resistant quasispecies. In lamivudine refractory patients, ETV is effective in suppressing viral replication and leading to histological improvement [136]. However, the emergence of resistance is more frequent in this setting rising from 6% at year 1 to 15, 36, 46, 51 and 57% at years 2, 3, 4, 5, and 6 of treatment, respectively [135]. This requires the presence of rtM204V and rtL180 M, but not rtM204I, and of an additional mutation at rtI169T, rtT184G, rtS202I, or rt M250V for resistance against ETV to emerge [137, 138].

### 5.4. Telbivudine (TBV)

Telbivudine is an analogue of thymidine which was approved for the treatment of CHB at a dose of 600 mg/day in 2006. It was initially shown to induce significantly greater virological and biochemical responses than LMV following 1-year treatment [139]. In the phase III trials in HBeAg positive Chinese patients, HBeAg seroconversion stood at 22 and 30% after 1 and 2 years of treatment, whilst HBV-DNA suppression at 60 and 56% for the same time periods. Similarly in HBeAg negative patients HBV-DNA drops were 88 and 82% at year 1 and 2, respectively [140, 141]. TBV was also shown to be superior to adefovir at suppressing HBV-DNA after 24 weeks of therapy [142].

Genotypic resistance against TBV has been reported at 4.4% and 21.6% at year 1 and 2 in HBeAg positive, whilst in HBeAg negative patients the figures were 2.7 and 8.6%, [141] rising further by the 3rd year and thereafter [143, 144]. The rtM204I alone or in combination with rtL180 M confers resistance to TBV and therefore cross-resistance to LMV also. Resistance is lower depending on HBV-DNA level status of the patient, whether HBeAg positive (<9 log copies/mL) or HBeAg negative (<7 log copies/mL) [145]. Patients with resistance can still be switched to ETV, as the mutation associated with resistance to this drug is rtM204V and not rtM204I. 

TBV treatment has been associated with elevation of creatine kinase and myopathy in some patients, [141, 142, 146] and peripheral neuropathy if given in combination with PegIFN [147].

### 5.5. Tenofovir (TFV)

Tenofovir disoproxil fumarate, an adenine analogue, was approved in 2008 for the treatment of CHB at a dose of 300 mg/day. TFV has been shown to be superior in terms of HBV-DNA suppression, HBeAg seroconversion, and ALT normalisation in HBeAg positive patients when compared to ADV [148, 149]. Undetectable levels of HBV-DNA (<400 copies/mL) were achieved in 76% and 93% of HBeAg positive and negative patients respectively; the figures for the ADV arm were 13% and 63% after 48 weeks of treatment [148]. Normalisation of ALT levels was seen in 68% versus 54%, histological improvement in 67% versus 12%, and HBsAg loss in 3.25 versus 0% in TFV and ADV treated patients, respectively [148]. Durability of response was almost 100% in both HBeAg positive and negative patients after 4 years of treatment [150–152]. More importantly, HBeAg loss after 4 years of treatment was recorded in 41% of patients and seroconversion in 29%, whilst the figures for HBsAg loss and seroconversion were 10% and 7.5%, respectively [151]. No resistance to TFV has been reported so far even after 5 years of followup [153].

TFV is generally well tolerated, but a few cases of Franconi syndrome have been reported [154]. Monitoring of serum creatinine and phosphorus levels is therefore advisable.

## 6. Other Drugs and Drugs in Development

Emtricitabine was initially approved for the treatment of HIV infection and shown to also have potency against HBV in both HBeAg positive and negative patients [155]. The drug is used in combination with tenofovir in HIV/HBV coinfected patients. Clevudine approved in South Korea and Philippines, was shown to be more potent than LMV at suppressing HBV-DNA [156]. However, its use has been associated with myopathy in the United States, and as both entecavir and tenofovir are more effective it seems unlikely that it would get approval in the West. Amdoxovir and ANA380 (LB80380) are two additional drugs which have shown promise in treatment naïve CHB patients or LMV resistant ones [157–159]. Bay 41–4109 is a drug that appears to interfere with core particle assembly, [160, 161] but its full potential has not been evaluated yet.

## 7. Conclusion

NA monotherapy treatments are effective in suppressing HBV replication, leading to HBeAg seroconversion, normalisation of ALT levels, improvement in histology and in some cases, even loss of HBsAg. Certain groups of patients have benefited tremendously from the use of NAs, such as those with decompensated cirrhosis and chronic HBV patients undergoing liver transplantation. Patients who do not respond to monotherapy treatment protocols may benefit from combination therapies, as has been the case in HIV treatment. However, although not reviewed here NAs in combination with each other or with IFN have not resulted in any additive or synergistic effects. For this to happen, it may be necessary to combine drugs with different modes of action in the future. Development of resistance is a drawback, as it undermines long-term suppression of viral replication, and any histological and biochemical improvement. However, the use of ETV and TFV, both of which have a high genetic barrier to resistance, has largely overcome this problem.

## 8. Hepatitis C Virus 

### 8.1. Virology

**Figure 3:**
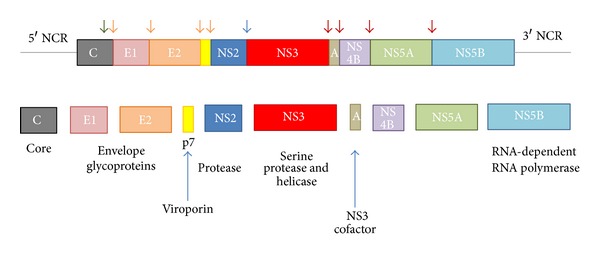
Genomic organisation of HCV showing the RNA, the polyprotein, and its products, following proteolytic cleavage. Cleavages by host signalases are shown in green and orange arrows, and those by virally encoded enzymes in blue (NS2/NS3) and brown (NS3/NS4A).

HCV is classified within the *Flaviviridae* family, under the genus of *Hepacivirus*. The virus particle has an outer envelope and an inner core, that in turn encloses the single stranded, positive sense RNA genome of the virus. The genome is 9.6 kb in length and contains a single ORF which is translated into a polyprotein of about 3000 amino acids long (Figure 3) [162]. The polyprotein is co- and posttranslationally cleaved to yield the structural viral proteins from its N-terminal end, whilst the C-terminal two thirds of the protein yield the nonstructural (NS) ones. The structural proteins include the core (C) protein responsible for the formation of the nucleocapsid and the two envelope glycoproteins E1 and E2. These and p7, which is thought to act as a viroporin, are processed by signal peptidases and a signal peptide peptidase resident within the ER lumen [163–165]. NS2 is an autoprotease, dimers of which in conjunction with the N-terminal end of NS3, allow its cleavage from the nonstructural portion of the polyprotein, whilst the remainder of the nonstructural proteins (NS3, NS4A, NS4B, NS5A, NS5B) are cleaved by NS3 which acts as a serine protease. NS4A is a cofactor which after its cleavage associates with NS3 assisting in the processing at the NS4B/NS5A and NS5A/NS5B junctions. NS4B and NS5A appear to play key roles in viral replication as explained below, whilst the NS5B constitutes the RNA-dependent RNA polymerase (RdRp) of the virus. The NS3 serine protease which also functions as a helicase, as well as the RdRp, have been widely studied and their 3D structure resolved by X-ray crystallography [166]. Moreover, the development of subgenomic replicons and full infection systems such as the JFH1 strain have permitted the testing of potential DAAs in vitro [167, 168]. Thus, DAAs that target these two enzymes, so crucial in the life cycle of the virus, have been developed as described below.

The ORF is flanked at either end by the 5′ and 3′ untranslated regions (UTRs), involved in translation and replication, respectively. The 5′ UTR forms a clover-leaf-like structure through intramolecular base-pairing known as the internal ribosome entry site, which recruits the translational ribosomal complex. Similarly the 3′ UTR has a triloop structure which recruits the replication complex for the synthesis of the full length replicative intermediate of negative sense [162].

### 8.2. Life Cycle

The virus circulates in blood in association with lipoproteins and is therefore referred to as a lipoviroparticle [169]. It binds to glycosaminoglycans and there follows a multistep attachment process mediated through receptor or coreceptor binding, which includes a number of molecules such as CD81, a tetraspanin and SRB1, which is involved in selective lipid uptake [170, 171]. Two junction proteins are also involved such as claudin-1 and occludin [172, 173]. More recently two receptor tyrosine kinases, EGFR and EphA2, and Niemann Pick C1-like 1 cholesterol absorption receptor have also been shown to be involved in HCV entry [174, 175]. The HCV particle is internalised by clathrin mediated endocytosis and following fusion of the viral envelope with early endosome membranes the capsid is released in the cytoplasm [176–178]. Uncoating and release of the RNA leads to the translation of the polyprotein and its processing to the various structural and NS components. The polyprotein is intimately associated with ER membranes so that E1 and E2 protrude into the ER lumen, whilst the NS3 through to NS5B, which constitute the replication complex, are associated with a network of membranes known as the membranous web. NS4B is instrumental in the formation of this web where RNA replication occurs. The core protein associates with lipid droplets and this association is very important in viral morphogenesis [179, 180]. Packaging of positive sense RNA genomes in core nucleocapsids is followed by budding through the ER membrane resulting in the acquisition of the outer viral envelope containing the E1/E2 heterodimers [181]. The NS2 and p7 proteins are thought to play a role in successful viral assembly also [182–184]. Virus particle maturation and release is intricately associated with the VLDL assembly pathway. Thus, apart from VLDL, lipoviroparticles contain LDL and apolipoproteins [169, 181, 185, 186].

Host factors are also important during viral replication and assembly, such as cyclophillin A which is thought to interact with NS5A [187, 188]. Another host factor is phosphatidylinositol 4 kinase III*α* (PI4KIII*α*) which is associated with the membranous web, once again through interaction with NS5A [189]. These factors constitute targets for antiviral therapy.

HCV has a considerable amount of genetic variation which has allowed the division of virus isolates into six genotypes (Gt1-6) and over 100 subtypes. In addition, the RdRp lacks proof-reading capacity resulting in the introduction of point mutations during RNA replication. Thus, the virus circulates within an infected individual as a swarm of closely related variants known as quasispecies. Gt identification is an important determinant in instituting antiviral treatment. Moreover, quasispecies analysis has revealed that at least 9% of treatment naïve patients have preexisting resistance mutations that would undermine antiviral therapy if instituted [190].

### 8.3. Standard of Care (SOC)

Currently the approved SOC involves the use of PegIFNa2a or −2b [191, 192] in combination with ribavirin (RBV), a nucleoside analogue of guanosine with broad antiviral activity [193]. PegIFN is administered once a week by intramuscular injection whilst RBV is taken orally twice a day. Weight based dosing of RBV has been shown to improve sustained virological responses (SVR), defined as undetectable HCV RNA 24 weeks after cessation of treatment of any duration [194]. RBV monotherapy has limited activity against HCV, but when used with PegIFN, it improves SVR significantly and has been the SOC for the last 10 years [195]. Depending on Gt, treatment duration lasts for 24 weeks for GT2/3 or 48 weeks for Gt1/4 leading to an SVR of 45% and 80%, respectively [192, 196]. A side effect of RBV use is the development of anaemia, which may compromise SVR if it becomes necessary to reduce the dosage.

Failure to achieve a >2 log decline in HCV RNA from baseline by week 12 is an indication for discontinuation of treatment, as the patient is unlikely to respond (null responders) [197]. On the other hand, patients with undetectable HCV RNA by week 4 (RVR, rapid virological response) are more likely to achieve SVR. Patients who experience a >2 log decline in HCV RNA by week 12 are said to have an early virological response (EVR), but if they fail to clear HCV RNA by week 24 are referred to as partial responders. Those who are negative for HCV RNA at the end of treatment but become positive during the 24-week followup are known as relapsers. Host factors including race, age, and fibrosis score, as well as HIV coinfection are known to be involved in poor responses to SOC treatment [191, 192, 198–201]. More recently, the discovery of a group of single nucleotide polymorphisms in the region of the IL28B gene (type III IFN) has widen the list of factors that can influence SOC outcome [202]. In particular, the CC genotype at the IL28B rs12979860 locus has proved the most powerful baseline predictor for up to 70% SVR in Gt1 patients as opposed to 25–30% with the CT or TT genotype.

### 8.4. DAAs

In 2011, two inhibitors of the NS3/4A serine protease were approved for the treatment of chronic HCV infection. These are telaprevir (TPV, Incivek) and boceprevir (BOC, Victrelis), both of which are active in patients with Gt1 infection. Both drugs are plagued with a number of adverse effects, but newer drugs in development are likely to overcome these problems, thus opening a new era in HCV treatment. The drugs are peptidomimetic, mimicking the polyprotein cleavage region, binding the active site of the enzyme, and thus preventing processing of the authentic substrate. There are two classes of these inhibitors which are the linear or covalent ketoamide derivatives and the macrocyclic (noncovalent) ones. TPV and BOC belong to the linear inhibitor group together with BI201335, whilst other inhibitors in development such as simeprevir (TMC435), danoprevir (ITMN191/R7227), vaniprevir (MK7009), GS-9256, and others are macrocyclic.

Although both TPV and BOC are potent protease inhibitors (PIs) when given as monotherapy, resistant variants are selected very quickly from the pool of quasispecies, compromising their antiviral effect. As a result both drugs have been approved for treble therapy that includes PegIFN and RBV, as PegIFN with a broad antiviral action is able to keep any variant in check.

### 8.5. Telaprevir (TPV)

TPV is effective in both treatment naïve and treatment-experienced patients with Gt1 infection as shown in the PROVE 1–3 trials [203–206]. These trials established that higher response rates were obtained when TPV was added to PegIFN and RBV (PR), that RVR occurred more frequently and there were lower rates of relapse. RBV was shown to be an integral part of the combination, and dose reduction to ≤600 mg/day did not compromise SVR rates [207]. In addition, the concept of response guided therapy whereby treatment was terminated early based on viral load measurement early in the course of treatment was introduced. The ADVANCE (phase 3) study compared 3 treatment arms that included 750 mg of TPV every 8 h together with PR for 8 weeks followed by additional weeks of PR (T8PR), TPV, and PR for 12 weeks (T12PR) or PR for 48 weeks (PR48).

Patients who had undetectable HCV RNA at 4 or 12 weeks (extended RVR, eRVR) received a total of 24 weeks of treatment, whereas those who did not were treated for 48 weeks. SVR rates in both TPV arms stood at 75% and 69% for the T12 and T8 groups, respectively, compared with 44% in the control group (PR). Relapse rates were 9% in the TPV arms and 28% in the control arm. 58% of the patients in the T12 arm achieved eRVR and of these 89% also attained SVR. Looking at the results from the T12 arm, SVR rates in African-Americans were 62% and in patients with bridging fibrosis or cirrhosis also 62%, as opposed to 25% and 33% in the PR arm, respectively. Discontinuation of treatment became necessary in 10% of the TPV treated patients as opposed to 7% of the controls, whilst rash and anaemia were the two main side effects of treatment with TPV. Grade 3 rash was seen in 5% of the TPV treated patients and 29% reported anorectal problems versus 7% of controls.

In the illuminate study, all patients received 12 weeks of TPV followed by 12 or 36 weeks of PR if eRVR was achieved. SVR rates were 92% and 88% for the 12 and 36 weeks of PR, respectively, thus indicating that the 24-week treatment regimen was noninferior to the 48 weeks [208].

TPV was equally effective in treatment experienced patients as demonstrated in the REALIZE trial [209]. The trial had 3 treatment arms; one with 4 weeks PR followed by addition of TPV (750 mg every 8 h) for 8 weeks and then 32 weeks of PR only (delayed start), a second arm with treble therapy for 12 weeks followed by 36 weeks of PR (simultaneous start) and the control arm entailing 48 weeks of PR treatment. There was no difference in results between the TPV arms. Therefore pooling the results together, SVR rates were 85.5% for previous relapsers, 56.5% for partial responders, and 31% for null responders versus 24%, 15%, and 5% in the control group, respectively. Presence of cirrhosis in null responders compromised SVR rates (14%), whilst bridging fibrosis or cirrhosis in partial responders had higher SVR rates (44%).

TPV is a potent inhibitor of CYP3A and therefore contraindicated in patients taking at the same time medicines that are highly dependent on CYP3A clearance, as high concentrations are associated with serious events. Other drugs include lovastatin, simvastatin and atorvastatin, PDE5 inhibitors, ergot products, and alfuzosin, as well as others. Drugs that induce CYP3A such as rifampicin and St John's wort should similarly not be used concurrently.

### 8.6. Boceprevir (BOC)

The phase 2b SPRINT-1 trial established that a 4-week lead-in with BOC and the inclusion of RBV were necessary for optimal SVR rates [210]. As in the case of TPV, two-phase 3 trials established that BOC was equally effective in Gt1 infected treatment-naïve and treatment-experienced patients [211, 212]. All patients in the SPRINT-2 trial received a 4-week lead-in of PR and then randomly assigned to receive either PR/placebo for a further 44 weeks, or PR/BOC 800 mg TID for a further 44 weeks, or PR/BOC for a further 24 weeks followed by PR for an extra 20 weeks if HCV RNA was still detectable between weeks 8–24 (RGT). Stopping rules were applied if patients were still HCV RNA positive at week 24. In both BOC arms SVR rates were almost identical, averaging 67.5% in nonblack patients as opposed to 40% in the control group. For black patients the figures were 53% and 42% for the 48-week BOC arm and the RGT group, respectively versus 23% for the controls. A reduction in HCV RNA of ≥1 log during the lead-in period was predictive of SVR in all groups. Relapse rates stood at 8.5% in the BOC arms versus 23% in the controls in the case of nonblack patients, whereas in black patients the relapse rate was similar in all groups ranging from 12–17%.

Adverse events necessitated discontinuation of treatment in 12–16% of patients in all 3 arms of the trial. Anaemia affected 49% of BOC-treated patients as opposed to 29% of controls, but even though dose reduction secondary to anaemia was more common in the BOC-treated patients (21 versus 13%), treatment discontinuation was rare (2 versus 1%). Additionally, dysgeusia was associated with BOC treatment.

The RESPOND-2 trial involved treatment-experienced patients (nonresponders or relapsers), [209] with treatment arms as in SPRINT-2 except for the RGT arm where the lead-in was followed by 32 weeks of treble therapy if HCV RNA was undetectable at week 8 or treble therapy for 32 weeks followed by 12 additional weeks of PR if HCV RNA was detectable at week 8. Treatment was discontinued in patients still positive for HCV RNA at week 12. SVR rates were 66% and 59% for the 44 weeks and RGT groups respectively versus 21% in controls. SVR was higher in relapsers than nonresponders, and once again a ≥1 log drop in HCV RNA at the end of the lead-in period entailed higher SVR rates at 79% and 73% for the 44 weeks and RGT groups, respectively. Even in patients with less than 1 log drop in HCV RNA the chances in the BOC arms of achieving SVR were higher at 33.5% versus 0% in controls. Discontinuation due to adverse events was very similar to SPRINT-2.

As with TPV, BOC is a potent inhibitor of CYP3A and therefore contraindicated for use with medications that are also dependent on CYP3A clearance. Also, its use should be avoided with medications that induce CYP3A to prevent loss of efficacy.

### 8.7. Resistance Associated Variants (RAVs)

Monotherapy with TPV or BOC selects preexisting resistant variants from the quasispecies pool within 1-2 weeks of starting treatment [213]. Such variants however are replication less competent. Cross-resistance between TPV and BOC has been reported [214]. Use of RBV appears to be important in minimising resistance at 2% in treble therapy regimens as opposed to 26% in RBV free use [200]. After stopping therapy, resistant viruses are overtaken once again by the wild type [214–216]. Amino acid substitutions that confer resistance and cross resistance between TPV and BOC, include R155 K, T54S/A, V36 M, and A156T.

### 8.8. NS3/4A DAAs in Clinical Testing

As already mentioned a number of other DAAs against the NS3/4A serine protease are at different stages of clinical testing. Simeprevir (TMC435) has shown efficacy when given with PR in both treatment naïve and treatment-experienced patients [217]. In vitro, it is active against all six genotypes, less so though against Gt3a [218, 219]. A side effect of treatment was reversible hyperbilirubinemia. The final results of the ASPIRE trial (phase-IIb) in Gt1 treatment-experienced patients were reported at the 2012 EASL meeting. SVR rates were 85%, 75%, and 51% versus 37%, 9%, and 19% in the 150 mg TMC435 arm versus controls for relapsers, partial, and null responders, respectively [220].

Danoprevir (ITMN191/R7227) is yet another potent macrocyclic PI, which has demonstrated high SVR rates [221]. Patients treated with high doses have experienced grade 4 ALT elevations. Therefore, to increase levels of the drug and restrict liver toxicity, the drug is coadministered with ritonavir and has been shown that in combination with PR is effective [222]. 

Other PIs giving promising results in phase-II studies include Asunaprevir (BMS-650032) and BI207127 [223].

### 8.9. RdRp Inhibitors

Two groups of such inhibitors have been developed so far: nucleoside and nonnucleoside inhibitors (NIs and NNIs). NIs are directed against the active site of the enzyme and are RNA chain terminators. The first such inhibitor to be developed was Valopicitabine which showed weak antiviral activity and was withdrawn due to gastrointestinal problems. More promising chain terminating drugs have since been developed which appear to have a very high genetic barrier to resistance and are active against all genotypes since the RdRp is highly conserved between genotypes [224]. In vitro studies have indicated that for resistance to occur three separate mutations are required [225]. On the other hand, NNIs interfere with RdRp function through binding to four allosteric sites, preventing the polymerase from assuming a functional conformation. NNIs binding to site 1 (thumb 1) include BI207127 and Tegobuvir (GS-9190), those binding to site 2 (thumb 2) include Filibuvir (PF-00868554) and VX-222, whilst those binding to sites 3 (palm 1) and 4 (palm 2) include ANA598 and ABT-333, respectively. NNIs have a low resistance barrier and some of them have limited clinical efficacy [224].

In the proton trial of the NI PSI-7977 combined with PR, SVR rates of more than 94% were reported in Gt1 infected patients, independently of predictors of poor IFN response, and similarly high rates of 96% in GT2 and 3 [226]. Recently, interim results from the ATOMIC study were reported showing eRVR rates of around 97% and SVR12 rates above 90% [227]. Mericitabine (RG7128) in combination with PR leads to high eRVR rates (>80%) but treatment with the drug for 8 or 12 weeks gave overall SVR rates not much different than the control arm, but analysing the results according to IL28B CC genotype, SVR rates ranged from 60–91 in the different treatment arms versus 58 in the PR group [228]. Treatment for 24 weeks with mericitabine, appears to be more effective with SVR12 rates of 58% versus 38% in the PR group [229]. 

NNIs have shown significant increases in eRVR rates in phase II studies, but SVR rates have been modest at around 50% in combination with PR [230]. Such inhibitors though may be useful when used in combination with others as described below.

### 8.10. NS4B

Clemizole hydrochloride has been shown to interfere with RNA binding to the arginine rich motif at the carboxyl end of the protein [231]. This agent is currently undergoing preclinical evaluation.

### 8.11. NS5A Inhibitors

The NS5A protein forms part of the viral replication complex, [232, 233] interacts with a number of cellular factors [234], and interferes with the innate immune response, [235] but has no enzymatic function. The protein has three domains involved with membrane binding (domain I), cyclophilin interaction (domain II) and virus assembly (domain III) [236]. Inhibitors have been identified which act against NS5A functions. One such compound is Daclatasvir (BMS-790052) which in a phase II study has shown high efficacy in treatment naïve Gt1 patients (SVR12 of 83–92% versus 24% in PR group), [237] as well as in null responders. 

Other NS5A inhibitors include GS5885, ACH-2928, and ABT-267, which in phase I studies have shown strong suppression of HCV RNA and are currently in phase II studies. Preliminary results with ABT-267 in combination with PR for 12 weeks followed by PR for 36 weeks achieved week 4 RVR in 74% and cEVR (complete early virological response) at week 12 in 87% of patients compared to 33% and 67% in the placebo group [238].

### 8.12. Quad Treatment

Use of two inhibitors in combination with PR has produced even more dramatic results. A study comparing a combination in treatment naïve Gt1 patients of a PI (GS-9256) and an NNI (Tegubovir, GS-9190) with or without RBV or PR for 4 weeks, achieved RVR rates of 100%, 38%, and 7% for the PI/NNI/PR, PI/NNI/RBV, and PI/NNI arms, respectively. Interestingly, the patients receiving quad treatment maintained HCV suppression (week 24), whilst 100% of treble therapy patients achieved cEVR by week 12 and maintained this response through week 24 [239, 240]. Viral breakthrough due to RAV, was responsible for the poor response in the two test agent arms. The ZENITH trial once again in treatment naïve Gt1 patients, using a combination of a PI (TPV) and an NNI (VX-222) with or without RBV, or with PR, indicated response rates in the PI/NNI/PR treated patients comparable to those seen with TPV/PR, without viral breakthrough. What is more, up to 50% of patients who had only 12 weeks of therapy achieved SVR12 rates of 82–93% [241]. Dual therapy failure was associated with viral breakthrough as a result of emergence of RAVs [242].

Quadruple therapy has also been attempted by combining the NS5A inhibitor Daclatasvir (BMS-790052) and the PI Asunaprevir (BMS-650032) with PR for 24 weeks, in Gt1 null responders [243, 244]. The quadruple combination achieved SVR in all treated patients, whilst the two drug arm showed a 55% viral breakthrough which affected all Gt1a patients and only one of the 1b patients.

SVR12 results were recently reported following combination treatment with ABT-450 (PI)/Ritonavir (ABT-450/r) and PR in Gt1 infected patients. The once daily dose arm of 200/100 mg of DAAs for 12 weeks followed by PR for 12–36 weeks yielded the highest SVR12 rate at 88%. 100% of patients in this arm had attained RVR (week 4), cEVR (HCV RNA undetectable at week 12), and EOT HCV RNA undetectable [245]. Speedy suppression of HCV RNA has also been reported with quadruple combination of Danoprevir/Ritonavir/PR at week 12 in the DAUPHINE trial, with average rates of HCV RNA levels <15 IU/mL in all Danoprevir/r arms of 89.5% as opposed to 45% in the control arm [246]. Finally, a combination of tegubovir, GS-9256 with PR resulted in an SVR rate of 98% after 16 weeks of treatment [247].

### 8.13. IFN-Free Regimens

The potent suppression of HCV RNA in combination treatments with PR raised the possibility of using IFN-free regimens for the treatment of chronic HCV infection, provided that the drugs used had a high genetic barrier to resistance. The first such study combined the NI Sofosbuvir (PSI-7977) and RBV for 12 weeks of therapy in Gt2 and 3 patients and resulted in 100% SVR12 (and also SVR24 in those that this information was available) [248]. A further trial employing Sofosbuvir and another analogue, PSI-938 led to the dropping of the latter due to hepatotoxicity problems. Dual therapy with Daclatasvir and Asunaprevir for 24 weeks in Gt1 Japanese patients who were either null responders or ineligible for or intolerant to IFN treatment, achieved SVR12 rates of 90% and 64%, respectively [249]. Daclatasvir in combination with Sofosbuvir, with or without RBV achieved SVR4 rates of 100% in Gt1, 91% in Gt2 and 3, independently of IL28B genotype. RBV did not have any advantage over dual therapy [250].

Treble therapy with the PI BI 201335, the NNI BI 207127, and with or without RBV produced an SVR12 rate of 68% in previously untreated Gt1 patients versus 38% in the RBV free combination, with favourable safety and efficacy among people with liver cirrhosis also. Response rates were higher in Gt1b and patients with the IL28B CC genotype. Viral breakthrough was also reported at an average of 11% between the treble treatment arms versus 29% in the dual therapy arm [251]. In cirrhotic patients, the treble therapy achieved SVR12 rates of 60% in Gt1a and 83% in Gt1b infected patients [252]. 

Quadruple therapy with ABT-450/r, the NNI ABT-072, and RBV was well tolerated and achieved an SVR24 rate of 91% in treatment naïve, noncirrhotic Gt1 infected patients with the IL28B CC genotype after 12 weeks of treatment [253]. A combination of GS-5885 (NS5A inhibitor), the NNI tegubovir, the PI GS-9451, and RBV achieved an SVR4 in 95% of patients treated for 12 weeks. Viral breakthrough and relapse was restricted to Gt1a infected patients [254].

### 8.14. Host Factor-Targeting Agents

Successful viral replication is dependent on a number of host factors such as cellular receptors, factors involved in replication, and scaffolding proteins involved in viral morphogenesis. Such factors constitute potential targets for antiviral treatment and the chances of viral resistance are quite low. 

Moreover, their use in combination with DAAs would theoretically reduce the dependence on IFN containing treatment regimens. However, some of these factors may be essential for normal host function and there may well be potential adverse effects.

#### 8.14.1. Cyclophilin Inhibitors

Cyclophilins are essential for viral replication, being part of the replication complex through interaction with NS5A [255]. Cyclosporine is an effective inhibitor of cyclophilins, but in view of its immunosuppressive effects through inhibition of calcineurin, cannot be used for treatment purposes. Nonimmunosuppressive alternatives have been developed such as Alisporivir (DEBIO 025) and SCY-635 [256, 257]. Alisporivir has shown broad genotype potency and selection of resistant variants has proved difficult [258]. Alisporivir in combination with PR in Gt1 patients achieved 76% SVR compared with 55% in the PR arm [259]. However, an ongoing trial had to be halted recently due to a number of pancreatitis cases, including one death.

#### 8.14.2. MiR-122 Inhibitors

 MicroRNAs are endogenous noncoding RNAs that are involved in regulation of gene expression by interfering with the translation and stability of target mRNAs. MiR-122 is a liver specific microRNA involved in HCV RNA replication [260]. Miravirsen (SPC3649) is a locked nucleic acid modified phosphorothioate antisense oligonucleotide complementary to the MiR-122 seed sequences [261]. The drug sequesters MiR-122 preventing it from binding to the 5′ UTR of HCV RNA and leads to long-lasting suppression of viraemia with no emergence of RAVs in chimpanzee studies [261]. Similar results have been reported in patients treated with weekly SC injections for 29 days, and followed up for 18 weeks [262].

#### 8.14.3. Entry Inhibitors

ITX-5061 is the first in class HCV entery inhibitor targeted against SR-B1 which is in clinical development [263]. Another potential entry inhibitor is Ezetimibe [175].

### 8.15. Conclusion

Antiviral treatment against chronic HCV infection has come a long way since the introduction of standard IFN as monotherapy. Over the years through the licensing of PegIFN in combination with RBV, and more recently the addition of PIs in treble treatment regimens has seen an increase in SVR rates well above 80% in difficult to treat patients such as those infected with Gt1. The future looks bright with the potential of using IFN sparing cocktails of DAAs able to achieve extremely high SVR rates. The success of such cocktails will be dependent on their ability to act pangenotypically, to prevent emergence of resistance, and to be adverse event free.

## 9. Hepatitis D Virus (HDV)

HDV is a defective RNA virus, more closely related to plant viroids than other human viruses, and dependent on HBV for its outer HBsAg containing envelope. In this respect, HDV infection can occur following exposure to both viruses concurrently (coinfection) or following exposure of somebody who is already a chronic HBV carrier (superinfection) [264]. In the latter case, there is increased likelihood of HDV infection becoming chronic also. Clinical disease in this scenario can be variable running either a benign course or causing severe liver disease which is more likely, with rapid progression to cirrhosis and HCC [265–267]. 

### 9.1. Virology

The HDV particle has a diameter of 36 nm and consists of an outer envelope containing all 3 forms of HBsAg. This surrounds the capsid which consists of hepatitis D antigen (HDAg) [268] and encloses the single stranded, circular RNA genome of the virus, which is of negative sense and approximately 1700 nt in length. The genome forms an unbranched rod-like structure due to intramolecular base-pairing that implicates more than 70% of its nucleotide sequence [269]. Unique features of this RNA are the presence of only one ORF which encodes the HDAg, the absence of a viral RNA polymerase and the presence of an RNA fold formed by about 85 nts, which acts as a ribozyme and is so essential during virus replication as explained below [270].

### 9.2. Replication

The virus possibly utilises the same cellular receptor as HBV due to their common HBsAg containing envelope. Uncoating occurs in the cytoplasm of the hepatocyte and the HDAg core is transferred to the nuclear pore, followed by delivery of the HDV RNA to the nucleoplasm. RNA transcription leads to the formation of a full length antigenomic strand which circularises and constitutes the template for genome synthesis, and of a subgenomic (0.8 kb), polyadenylated mRNA that encodes for HDAg. RNA synthesis occurs in the nucleus and implicates the nucleolus in antigenomic strand synthesis through the use of RNA polymerase I, whilst genomic strand synthesis occurs in the nucleoplasm and utilsises RNA polymerase II [271]. During RNA synthesis, both the genomic and antigenomic templates are employed in a rolling circle mechanism to produce concatemers of multiple full length RNA copies, which are cleaved by the ribozyme fold present in both forms of RNA to unit lengths, which then circularize [272]. There are two forms of HDAg which are known as small (S-HDAg, 195aa long) and large (L-HDAg, 214a long). These are encoded by the subgenomic mRNA, [273] and the L-HDAg arises through an RNA editing event that involves adenosine deaminase-1 that converts the termination codon for the S-HDAg to one for tryptophan. Thus, the L-HDAg has an extension of 19 further aa [269, 273]. The two forms of HDAg have unique functions in that the S-HDAg promotes replication whilst the L-HDAg suppresses replication and promotes viral morphogenesis [274].

### 9.3. Treatment

Treatment regimens for chronic HDV should target the helper HBV also for optimal results. Standard and recombinant interferons were thus first used for the treatment of chronic HDV infection but proved disappointing, in that after 12 months of treatment virologic responses were largely not sustainable [275, 276]. More recent use of PegIFN has produced variable SVR rates (17–43%) which are defined as negative HDV-RNA 6 months after cessation of treatment. The number of patients included in such trials was relatively small compared to the numbers enrolled in similar HBV and HCV trials and SVR rates may have been affected by heterogeneous baseline clinical, demographic, and virological parameters. PegIFN monotherapy has attained SVR rates of 17–25%, [277, 278] with the exception of one trial involving 14 patients which reported an SVR rate of 43% [279]. Addition of RBV to the treatment regimen had no additive effect [277, 280]. Similarly, PegIFN in combination with LMV or ADV did not increase SVR rates, whilst monotherapy with either LMV or ADV achieved SVR rates of 12%, and 0% respectively [281, 282].

Treatment regimens which suppress HBV DNA, although desirable, are unlikely to effect HDV clearance as HBsAg may still be provided by integrated HBV DNA sequences. HBsAg clearance has proved a difficult endpoint to achieve following antiviral treatment. Novel treatments targeting HDV replication are restricted as a result of the limited coding capacity of the genome to just one protein, namely, HDAg, and the complete absence of virally encoded enzymes. However, HDAg undergoes posttranslational modification through acetylation, methylation, and phosphorylation for optimal function. Interference with these processes is possible and may undermine virus replication. Indeed prenylation of the L-HDAg is essential for viral assembly and secretion [283]. Prenylation inhibitors have been shown to be effective in clearing HDV RNA in a mouse model [284]. With evidence from epidemiological studies that HDV prevalence remains unchanged, and in fact is some European countries may be on the increase as a result of immigration, there should be renewed efforts for novel drug development.
